# Differential placental methylation and expression of *VEGF, FLT-*1 and *KDR* genes in human term and preterm preeclampsia

**DOI:** 10.1186/1868-7083-5-6

**Published:** 2013-04-26

**Authors:** Deepali P Sundrani, Umakar S Reddy, Asmita A Joshi, Savita S Mehendale, Preeti M Chavan-Gautam, Anandwardhan A Hardikar, Giriraj R Chandak, Sadhana R Joshi

**Affiliations:** 1 Department of Nutritional Medicine, Interactive Research School for Health Affairs, Bharati Vidyapeeth University, Pune, 411043, India; 2 Department of Obstetrics and Gynecology, Bharati Medical College and Hospital, Bharati Vidyapeeth University, Pune, 411043, India; 3 Centre for Cellular and Molecular Biology, Council of Scientific and Industrial Research (CSIR), Hyderabad, 500007, India; 4 Diabetes and Islet-biology Group, NHMRC Clinical Trials Centre, The University of Sydney, Camperdown, NSW, 2050, Australia

## Abstract

**Background:**

Preeclampsia, a pregnancy complication of placental origin is associated with altered expression of angiogenic factors and their receptors. Recently, there is considerable interest in understanding the role of adverse intrauterine conditions in placental dysfunction and adverse pregnancy outcomes. Since we have observed changes in placental global DNA methylation levels in preeclampsia, this study was undertaken to examine gene promoter CpG methylation and expression of several angiogenic genes.

We recruited 139 women comprising, 46 normotensive women with term delivery (≥37 weeks), 45 women with preeclampsia delivering preterm (<37 weeks) and 48 women with preeclampsia delivering at term. Expression levels and promoter CpG methylation of *VEGF*, *FLT*-1 and *KDR* genes in placentae from respective groups were determined by Taqman-based quantitative real time PCR and by the Sequenom® EpiTYPER™ technology respectively.

**Results:**

We observed several differentially methylated CpG sites in the promoter regions of *VEGF*, *FLT*-1 and *KDR* between the normotensive and preeclampsia groups. We specifically observed hypomethylated CpGs in the promoter region and an increased expression of *VEGF* gene between term and preterm preeclampsia. However, mean promoter CpG methylation could not account for the higher expression of *FLT*-1 and *KDR* in preterm preeclampsia as compared to normotensive group.

**Conclusions:**

Our data indicates altered DNA methylation patterns in the *VEGF*, *FLT*-1 and *KDR* genes in preeclampsia as compared to the normotensive group, which could be involved in the pathophysiology of preeclampsia. Hypomethylation of *VEGF* promoter and consequent upregulation of *VEGF* mRNA levels could be a compensatory mechanism to restore normal angiogenesis and blood flow in preterm preeclampsia. This study suggests a role of altered DNA methylation in placental angiogenesis and in determining adverse pregnancy outcomes.

## Background

Preeclampsia (PE) originates in the placenta and involves inadequate cytotrophoblast invasion, maternal endothelial dysfunction and altered expression of angiogenic and anti-angiogenic factors, which ultimately leads to various clinical manifestations [1]. Vasculogenesis and angiogenesis are considered to be central processes in the development of the placenta and are mainly controlled by vascular endothelial growth factor (*VEGF*). Altered levels of *VEGF* and its receptors can disrupt angiogenesis, leading to placental insufficiency and endothelial dysfunction seen in PE [2]. *VEGF* exerts its biological effects through two high-affinity tyrosine kinase receptors namely, vascular endothelial growth factor receptor-1 (*VEGFR-1*)/fms-like tyrosine kinase-1 (*FLT*-1), and vascular endothelial growth factor receptor-2/kinase insert domain containing receptor (*KDR*). *FLT*-1 interactions with *VEGF* are critical for invasion and pseudo-vasculogenesis while *KDR* is a major mediator of mitogenic and angiogenic processes that enhance permeability and endothelial survival [3].

A number of studies have examined the mRNA levels of different angiogenesis regulating factors in placentae from PE patients although results are inconsistent. Some studies report increased *VEGF* expression [4-7], while others report reduced expression [8-10] in preeclamptic women. Other studies found no difference in *VEGF* expression [11-13]. Similarly expression of *FLT*-1 [14-16] and *KDR*[11,16,17] have also been examined. Very few studies have simultaneously examined the expression of *VEGF* and both its receptors in the human placenta in PE [18,19]. Further, most of the reported studies have been carried out on small sample sizes and are limited by the broad range of gestational ages.

Healthy placental development involves spatio-temporally programmed gene expression patterns and any alteration in this process may compromise placental function [20-22]. Under suboptimal uterine conditions, normal methylation of DNA is disrupted, thereby altering gene expression and preventing normal growth [23]. The placenta serves as the interface between the mother and fetus. A number of environmental factors such as diet, smoking, stress, behavior and assisted reproductive techniques have been reported to influence the methylation and expression of various genes in the human placenta as well as from other animals [24,25]. We have earlier observed increased homocysteine and oxidative stress levels in PE [26] and shown association of alterations in placental global DNA methylation levels with homocysteine levels and blood pressure [27]. The objective of this study was to examine the methylation of the promoters of the *VEGF*, *FLT-1* and *KDR* genes and their mRNA levels in placentae from women diagnosed with PE as compared to normal pregnancies. We further investigated whether the gene expression levels were related to promoter CpG methylation levels. To the best of our knowledge, no study to date has investigated the methylation patterns of the angiogenic gene *VEGF* and its receptors in the human placentae in PE.

## Results

### Maternal and neonatal characteristics

The maternal and neonatal characteristics are given in Table 1. All the women recruited in the study had similar age, income and education. The frequency of consumption of foods rich in folic acid, vitamin B_12_ and omega-3 fatty acids was similar in normotensive and both the PE groups as reported by us earlier [26,28]. The body weight and gestational age at delivery were significantly lower (*P* <0.05) in women with PE who delivered preterm. The maternal systolic and diastolic blood pressures were significantly higher in the term and preterm PE groups compared to the normotensive group. Newborn weight was significantly reduced (*P* <0.01) in the term PE group as compared to the normotensive group. The newborn weight, height, head circumference, chest circumference and APGAR score at 1 minute and 5 minutes were significantly lower (*P* <0.01) in the preterm preeclampsia group compared to the normotensive and term PE groups.

**Table 1:** Maternal and neonatal characteristics

	**Normotensive**	**Term PE**	**Preterm PE**
	**(n = 46)**	**(n = 48)**	**(n = 45)**
**Maternal characteristics**			
Age, yrs	22.9 ± 3.2	23.1 ± 2.9	23.9 ± 4.2
Weight, kg	52.1 ± 8.2	52.0 ± 11.2	47.7 ± 6.6*^1^
Height, cm	152.7 ± 4.9	152.3 ± 7.2	150.7 ± 5.4
Body mass index, kg/m^2^	22.3 ± 3.4	22.4 ± 4.8	20.9 ± 2.7
Gestation, wks	39.0 ± 1.2	38.6 ± 1.1	34.2 ± 1.9**^1^
Education, grade	9.2 ± 4.0	9.8 ± 3.5	9.4 ± 3.9
Income, Rs	6,191.5 ± 4,524.8	5,844.4 ± 3,285.3	6,258.5 ± 5,183.0
Systolic blood pressure, mmHg	123.4 ± 7.2	152.2 ± 16.0**	154.3 ± 15.8**
Diastolic blood pressure, mmHg	78.3 ± 6.0	100.8 ± 11.5**	103.0 ± 13.3**
Parity, %			
Nulliparous	42.6	68.9	58.5
Multiparous	57.4	31.1	41.5
Mode of delivery, %			
Normal delivery	70.2	78.3	45.2
Caesarean section	29.8	21.7	54.8
**Neonatal characteristics**			
Gender of fetus, %			
Male	54.3	51.1	42.9
Female	45.7	48.9	57.1
APGAR score (1 minute)	7.7 ± 1.2	7.6 ± 1.2	6.6 ± 1.8**^1^
APGAR score (5 minute)	8.6 ± 0.7	8.8 ± 0.7	8.1 ± 1.0**^1^
Weight, kg	2.9 ± 0.3	2.7 ± 0.5**	1.9 ± 0.5**^1^
Length, cm	48.1 ± 2.6	48.1 ± 2.4	43.8 ± 4.4**^1^
Head circumference, cm	33.9 ± 1.3	33.3 ± 1.7	30.3 ± 3.0**^1^
Chest circumference, cm	32.3 ± 1.4	31.7 ± 2.1	27.1 ± 2.9**

Values given are mean ± SD, unless stated otherwise. PE, preeclampsia. *P <0.05; **P <0.01 when compared to term; ^1^*P* <0.01 when compared to term PE.

### Promoter CpG methylation of *VEGF*, *FLT*-1 and *KDR* genes

**Figure 1:**
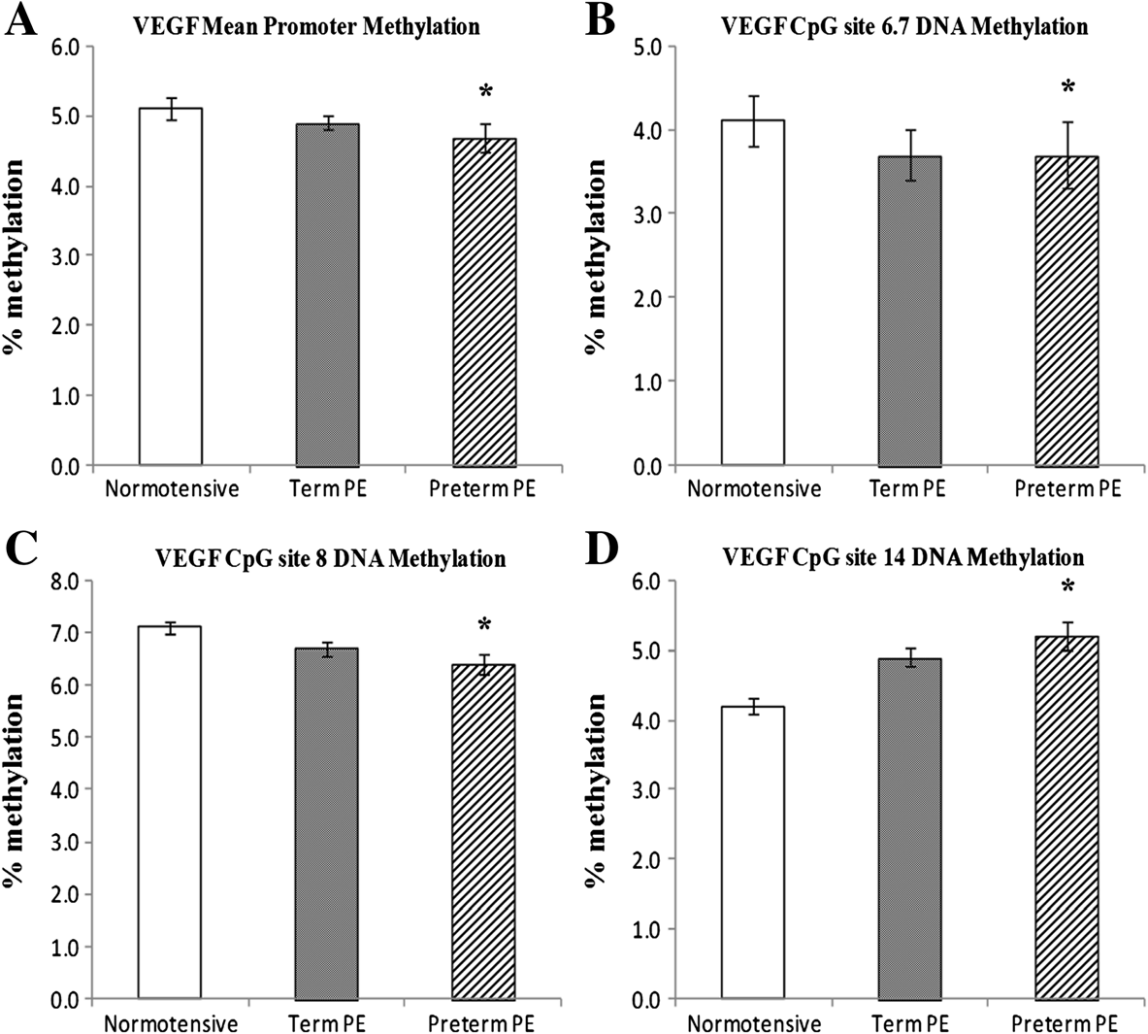
**Mean promoter methylation levels and differentially methylated sites in the *VEGF *promoter: (A) *VEGF *Mean Promoter; (B) *VEGF* CpG site 6.7; (C) *VEGF *CpG site 8; (D) *VEGF *CpG site 14.** *P <0.05 compared to normotensive group. PE, preeclampsia.

**Figure 2:**
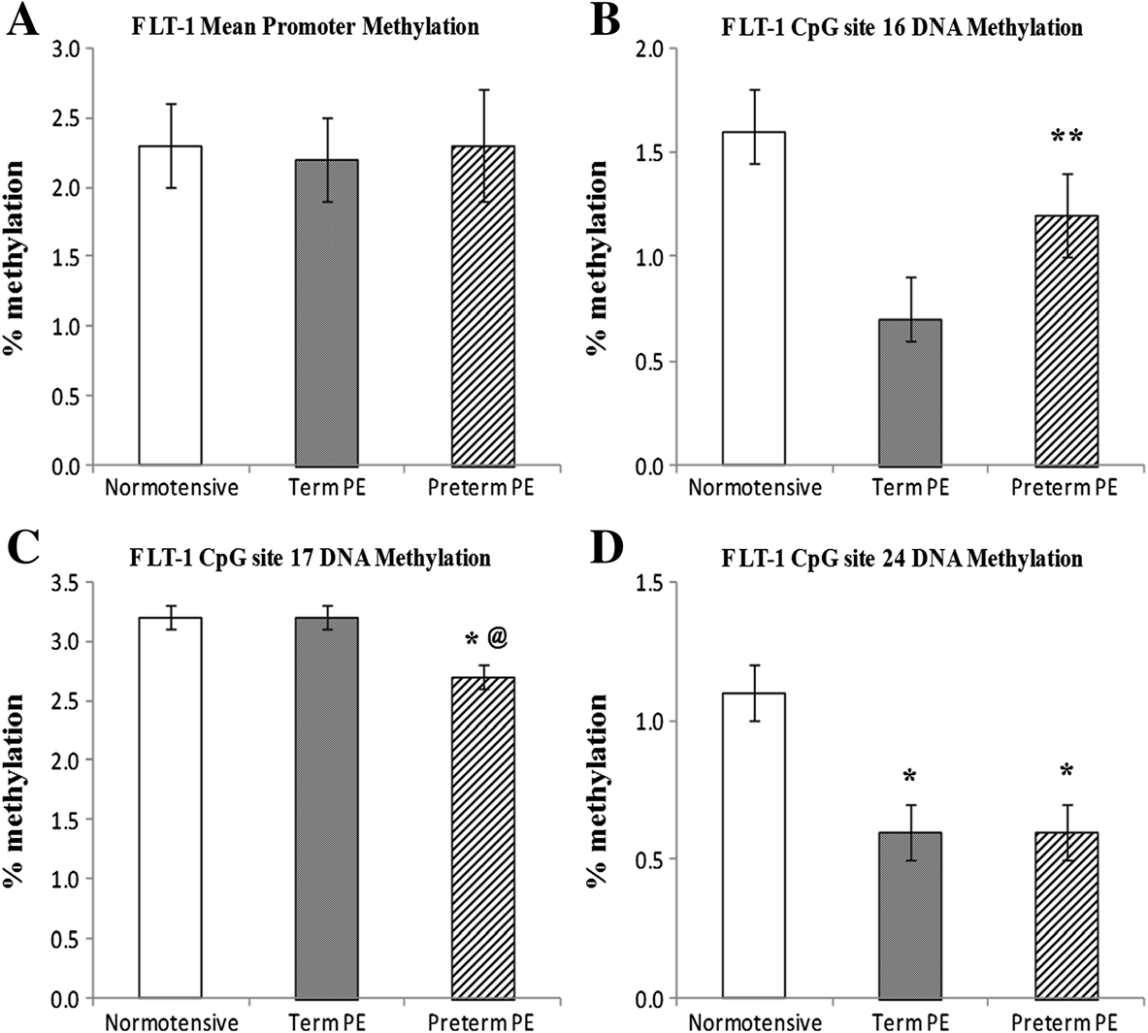
**Mean promoter methylation levels and differentially methylated sites in the *FLT*-1 promoter: (A) *FLT*-1 Mean Promoter; (B) *FLT*-1 CpG site 16; (C) *FLT*-1 CpG site 17; (D) *FLT*-1 CpG site 24.** *P <0.05 and **P <0.01 compared to normotensive group; ^@^P <0.05 compared to the term PE group. PE, preeclampsia.

**Figure 3:**
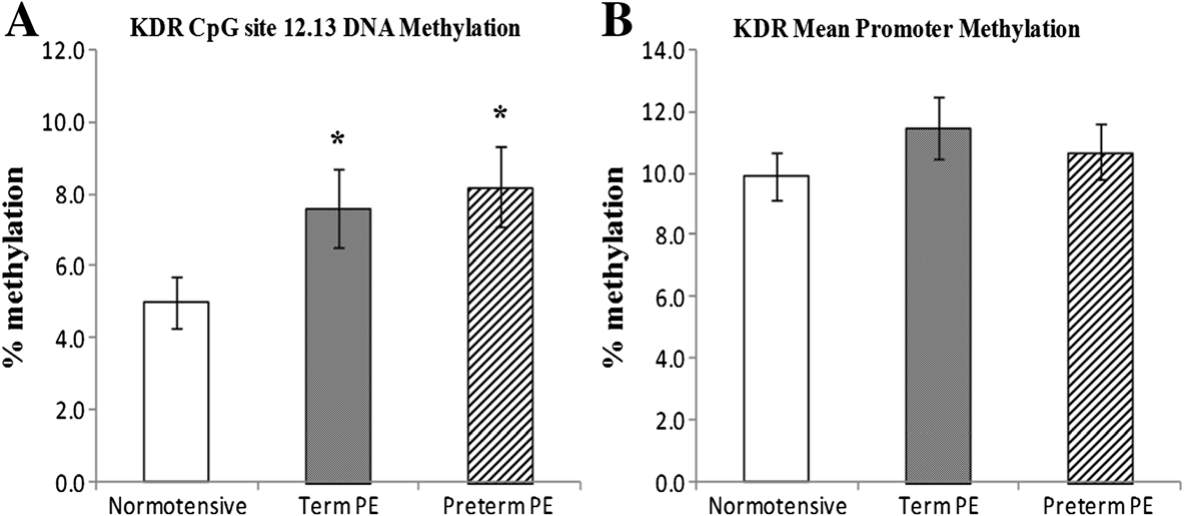
**Mean promoter methylation levels and differentially methylated sites in the *KDR *promoter: (A)* KDR* CpG site 12.13; (B) *KDR *Mean Promoter.** *P <0.05 and **P <0.01 as compared to normotensive group. PE, preeclampsia.

We analyzed cytosine methylation at 23 CpG sites in the *VEGF* promoter region, 30 CpG sites in the *FLT*-1 promoter region and 37 CpG sites in the *KDR* promoter. The mean percent methylation at all CpG sites in the promoter regions of the three genes are given in Additional file 1. The mean percent methylation at the differentially methylated CpG sites in the promoter regions of the three genes are given in Table 2. The mean methylation level of the *VEGF* promoter was significantly lower (*P* <0.05) in the preterm PE group compared to the normotensive group. The mean methylation at the CpG site 6.7 (*P* <0.05) and CpG site 8 (*P* <0.01) was significantly reduced, whereas that at CpG site 14 was significantly higher (*P* < 0.05) in the preterm PE group compared to the normotensive group (Figure 1). The mean methylation level of the *FLT*-1 gene promoter was similar between the groups. The mean methylation at CpG site 16 in the *FLT*-1 promoter region was significantly reduced (*P* <0.01) in the term PE group as compared to the normotensive group, while mean methylation at CpG site 17 was significantly reduced (*P* <0.05) in the preterm PE group compared to the normotensive group. Further mean methylation at the CpG site 24 was significantly reduced (*P* <0.05) in both the term and the preterm PE group compared to the normotensive group (Figure 2). There was no significant difference in mean methylation level of the *KDR* gene promoter between the three groups. We observed significantly higher mean methylation at CpG site 12.13 in the *KDR* gene promoter region in term PE (*P* <0.05) and preterm PE (*P* <0.01) group compared to the normotensive group (Figure 3). Further, several transcription factor binding sites were predicted in the promoter region of *VEGF*, *FLT*-1 and *KDR* genes (Additional file 2).

**Table 2:** **Mean percent methylation levels at the differentially methylated CpG sites in the promoter region of the *VEGF*, *FLT*-1 and *KDR *genes**

	**Percent methylation (%)**		
**CpG site**	**Mean ± standard error**		
	**Normotensive**	**Term PE**	**Preterm PE**
***VEGF***			
**Mean promoter**	5.1 ± 0.1	4.9 ± 0.1	4.7 ± 0.2*
**CpG - 6.7**	4.2 ± 0.2	3.7 ± 0.2	3.5 ± 0.4*
**CpG – 8**	7.1 ± 0.1	6.8 ± 0.1	6.4 ± 0.2**
**CpG – 14**	4.2 ± 0.3	4.9 ± 0.3	5.2 ± 0.4*
***FLT*-1**			
**CpG – 16**	1.6 ± 0.2	0.8 ± 0.2**	1.2 ± 0.2
**CpG – 17**	3.2 ± 0.1	3.2 ± 0.1	2.7 ± 0.1*^1^
**CpG – 24**	1.1 ± 0.1	0.7 ± 0.1*	0.6 ± 0.1*
***KDR***			
**CpG – 12.13**	4.9 ± 0.7	7.6 ± 1.1*	8.2 ± 1.1*

Values given are mean ± SE. PE, preeclampsia. *P <0.05; **P <0.01 compared to term; ^1^*P* <0.05 compared to term PE.

### *VEGF*, *FLT*-1, and *KDR* gene expression levels in placenta

**Figure 4:**
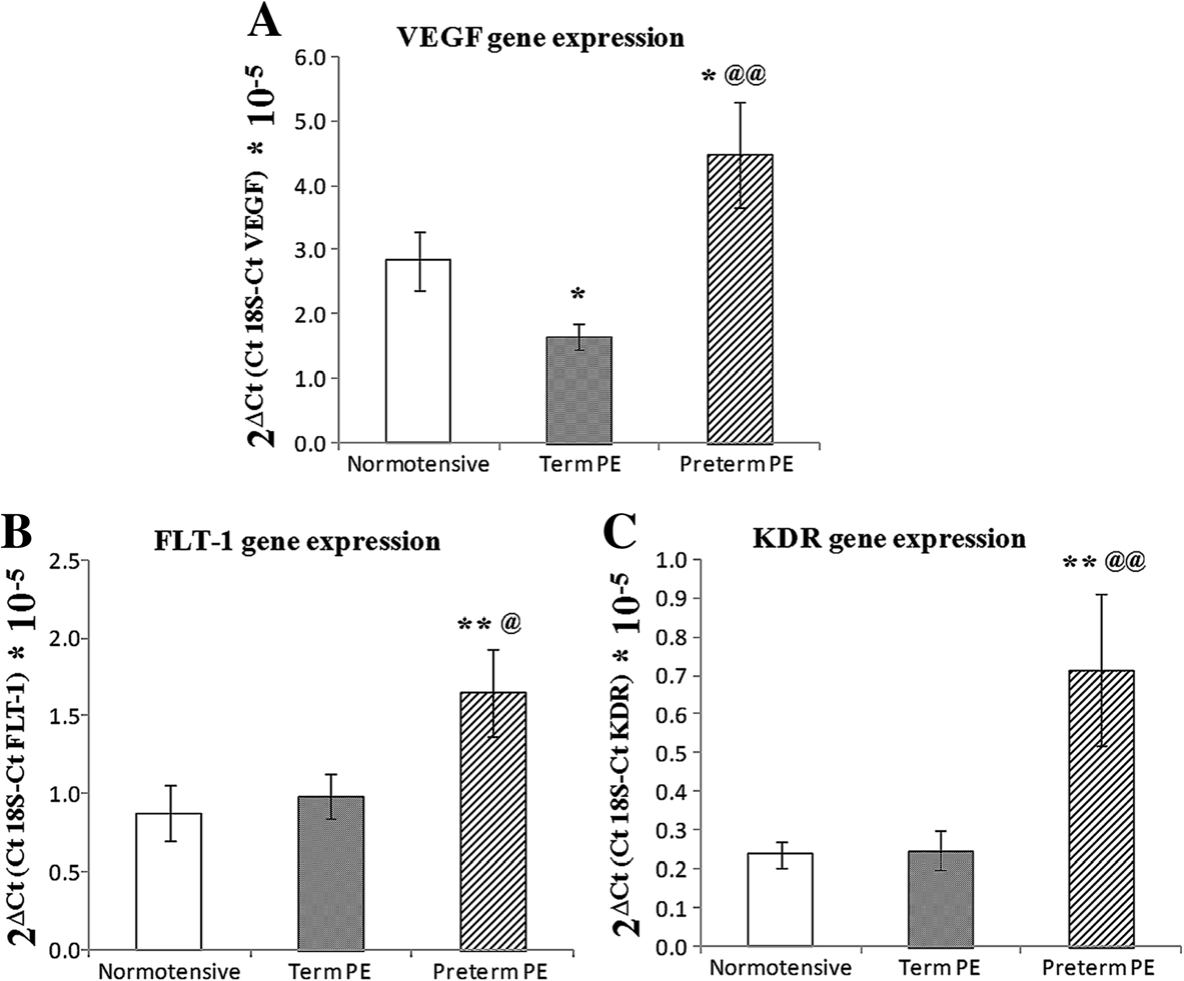
**Gene expression levels in normotensive and preeclamptic groups: (A) *VEGF *Gene Expression; (B) *FLT*-1 Gene Expression; (C) *KDR *Gene Expression.** *P <0.05 and **P <0.01 when compared to normotensive group; ^@^*P* <0.05 and ^@@^*P* <0.01 when compared to the Term PE group. PE, preeclampsia.

The placental gene expression levels of *VEGF* were significantly lower in the term PE group than in the normotensive group (*P* <0.05), while the levels were significantly higher (1.6-fold) in the preterm PE group compared to the normotensive group. The gene expression levels of *FLT*-1 and *KDR* were comparable in the normotensive and term PE groups but were higher (1.9-fold for *FLT*-1 and 3.0-fold for *KDR*) in the preterm PE group compared to the term PE (*P* <0.05 for *FLT*-1 and *P* <0.01 for *KDR*) and the normotensive group (*P* <0.01) (Figure 4).

### Association between CpG methylation and gestation

Mean promoter methylation of the *VEGF* gene was negatively (n = 24, *r* = −0.461, *P* = 0.018) associated with gestation in the term PE group but not in the normotensive and preterm PE groups. Further CpG site 6.7 in the *VEGF* promoter region was negatively associated with both the term PE (n = 24, *r* = −0.399, *P* = 0.044) and preterm PE gestations (n = 19, r = −0.463, *P* = 0.034). Mean promoter methylation in the *FLT*-1 gene was not associated with gestation in any of the groups. However, CpG site 16 showed a positive association with preterm PE gestation (n = 30, *r* = 0.420, *P* = 0.020). There were no associations between methylation and gestation in the *KDR* gene promoter region.

### Associations between CpG methylation and gene expression

The mean methylation at CpG site 6.7 in the *VEGF* promoter region was positively associated with *VEGF* gene expression levels in the term PE group (n = 36, *r* = 0.331, *P* = 0.049). The mean methylation at CpG site 16 in the *FLT*-1 promoter region was negatively associated with *FLT*-1 gene expression levels in the normotensive group (n = 32, *r* = −0.392, *P* = 0.026). In contrast mean methylation at CpG site 24 in the *FLT*-1 promoter region was positively associated with *FLT*-1 gene expression in the preterm PE group (n = 30, *r* = 0.434, *P* = 0.017). Further the mean methylation at CpG site 12.13 in the *KDR* promoter region was negatively associated with *KDR* gene expression in the term PE group (n = 30, *r* = −0.369, *P* = 0.045).

## Discussion

In this study, we examined the CpG methylation of *VEGF*, *FLT*-1 and *KDR* gene promoters and their expression in human placentae. These genes encode important proteins that determine placental angiogenesis. This study shows several interesting findings: 1) some CpG sites in the promoter regions of these genes showed differential methylation between the normotensive, term and preterm PE groups; 2) mean promoter methylation in the *VEGF* gene was significantly lower in the preterm PE group compared to normotensive, while it was comparable between normotensive and term PE group; 3) *VEGF* expression was significantly higher in the preterm PE compared to the normotensive group, while it was lower in the term PE group; 4) although mean methylation in the *FLT*-1 and *KDR* promoters was similar between the three groups, *FLT*-1 and *KDR* gene expression was significantly higher in the preterm PE group compared to the term PE and the normotensive group; 5) mean methylation at the *VEGF* promoter and methylation at some differentially methylated CpG sites in the *VEGF* and *FLT*-1 promoters was associated with term and preterm gestations, and 6) mean methylation at some differentially methylated CpG sites in the *VEGF*, *FLT*-1 and *KDR* promoters was associated with their gene expression levels.

DNA methylation is an important epigenetic mechanism of gene regulation. In the *VEGF*, *FLT*-1 and *KDR* promoter regions, some CpG sites were differentially methylated between the normotensive and PE groups. These results indicate possible involvement of altered DNA methylation patterns in these genes, which could be involved in the pathophysiology of PE. In humans, DNA methylation is mediated by DNA methyltransferases that are responsible for *de novo* methylation and maintenance of methylation patterns during replication. There is abundant evidence that suggests that DNA methylation patterns can be altered as a component of disease pathogenesis [29,30]. However, further studies are needed to determine the functional relevance of these findings in the pathophysiology of PE.

Alterations in methylation status within promoter regions can affect gene expression and hence the phenotype [31]. Our results show an increase in *VEGF* mRNA in preterm PE compared to term PE and normotensive placentas. It has been suggested that in severe PE, higher levels of placental hypoxia inducible transcription factors upregulate *VEGF* expression [32]. Upregulation of *VEGF* in preterm the PE placenta is suggestive of a compensatory mechanism attempting to normalize angiogenesis and blood flow. Furthermore, the *VEGF* promoter (mean promoter methylation) was significantly hypomethylated in the preterm PE group and this may be responsible for the increased expression of *VEGF* observed in this group. These results are consistent with other reports that suggest an inverse relationship between promoter CpG methylation and gene expression and indicate epigenetic control of *VEGF* expression in preterm PE [33,34]. It is also possible that the CpG sites 6.7, 8 and 14, which are differentially methylated in the preterm PE group compared to the normotensive group, may be involved in the upregulation of *VEGF* mRNA levels.

Although the mean methylation of the *VEGF* promoter was comparable between the normotensive and term PE group, the gene expression levels were significantly lower in the term PE group compared to the normotensive group. Generally, the pathology of preterm PE is regarded as more severe compared to term PE. Previous studies have suggested the existence of different subsets of PE and that pathophysiologic mechanisms may contribute differently to the development of preterm versus term PE [35,36]. It is likely that since term PE is less severe compared to preterm PE, the compensatory increase in *VEGF* mRNA levels is not observed, neither is there a difference in the promoter methylation levels. The observed decrease in *VEGF* expression in the term PE may be due to alternative pathways of gene expression regulation such as alterations in transcription factor expression or histone modifications as a consequence of the pathology. Some previous studies have also shown reduced placental *VEGF* mRNA levels at term in the PE compared to the normotensive group [9,19].

Mean methylation of the *FLT*-1 and *KDR* promoters was comparable between groups, yet the mRNA levels of *FLT*-1 and *KDR* were significantly higher in the preterm PE group compared to the other two groups. Although there was no difference in the mean promoter methylation in the *FLT*-1 gene promoter, CpG site 17 was significantly hypomethylated in the preterm PE group compared to the normotensive and term PE group. This site may be important in influencing the *FLT*-1 expression in preterm PE, however further studies are needed to determine the exact role of this site in the regulation of *FLT*-1 expression. Further *KDR* expression in preterm PE may not be mediated through DNA methylation changes but through other factors affecting gene expression such as transcription factors, mRNA stability and histone modifications. Modulation of factors affecting gene expression by intracellular signals in different physiological states is well established. The opposite trends in *VEGF*, *FLT*-1 and *KDR* mRNA levels and the difference in epigenetic patterns between these two groups provide further support for the existence of differences in pathology between term and preterm PE.

Our previous studies have established that omega 3 fatty acids and micronutrients (folic acid and vitamin B_12_) are interlinked in the one carbon cycle [37,38]. Further, we have also reported decreased omega 3 fatty acids and increased plasma homocysteine levels in preeclamptic mothers [26]. Since these pathways are major determinants of the methylation potential of the cell, our results could affect DNA methylation patterns, thereby influencing regulation of vital genes [39].

Further we attempted to understand the functional relevance of methylation changes by investigating whether the differentially methylated CpG sites in *VEGF*, *FLT*-1 and *KDR* gene promoters were located within transcription factor binding sites. Although, few transcription factor binding sites were predicted in close proximity of the differentially methylated CpG sites in promoter regions of all three genes, it is difficult to assign importance to these changes. Further studies are needed to understand the role of methylation at these CpG sites in regulating the gene expression through transcription factor binding.

We observed that mean methylation of the *VEGF* promoter and some differentially methylated sites in the *VEGF* and *FLT*-1 promoters were associated with term and preterm gestations. CpG 6.7 methylation in the *VEGF* promoter was negatively associated with term and preterm PE gestations. Methylation of CpG 16 in the *FLT*-1 promoter was positively associated with preterm PE gestation. Human pregnancy comprises a complex series of differentiation and growth processes that are spatio-temporally regulated [23]. We have previously reported gestation-dependent changes in placental global DNA methylation [20]. Novakovic *et al*. (2009) have reported changes in promoter CpG methylation (both increase and decrease in methylation) with gestation in the placenta [40]. In addition to CpG sites that consistently change over gestation, they also report the existence of CpG sites that show inter-individual variability within each gestational age and suggest that such variability could be attributed to cumulative differences in environmental exposure.

Our results show that mean methylation of some of the differentially methylated sites in the *VEGF, FLT*-1 and *KDR* promoters were positively or negatively associated with their gene expression levels. Although hypermethylation is generally associated with suppression of gene expression, recent studies have also reported a positive association between methylation and gene expression [41-43].

## Conclusions

To summarize, the CpG methylation patterns and expression of *VEGF* differs between the preterm PE and term PE group and could be causally related to the differences in pathology of term and preterm PE. We have previously reported altered one-carbon metabolism, hyperhomocysteinemia and increased oxidative stress in PE, which are known to influence DNA methylation patterns. Thus, our results could be attributed to these changes in the intrauterine environment. However, it is unclear whether the observed differences are a cause or effect of the underlying pathophysiology. Nevertheless, this study reiterates that CpG methylation is dynamic and influenced by the intrauterine environment. Further, as seen in the case of the *VEGF* gene, DNA methylation changes could account for the alterations in gene expression, and the ability to induce compensatory mechanisms to circumvent adverse pregnancy outcome. This study also highlights that DNA methylation may not explain all gene expression changes, and other mechanisms of gene expression regulation also come into play. The role of CpG methylation, in other regions in the gene also cannot be ruled out, since it has also been suggested that methylation changes in the gene body could also affect expression [44]. This study is the first account of CpG methylation changes in the *VEGF*, *FLT*-1 and *KDR* gene promoters in pregnancies complicated by PE.

## Methods

### Subjects

This study was conducted at the Department of Obstetrics and Gynecology, Bharati Hospital, Pune with the understanding and consent of each subject and was approved by the Bharati Vidyapeeth Medical College Institutional Ethical Committee. A total number of 139 pregnant women with singleton pregnancy were recruited for this study. Of these, 46 women had normotensive pregnancies and delivered at term, 93 women had PE during pregnancy, of which 45 delivered preterm (<37 weeks), while 48 delivered at term (≥37 weeks). Women were excluded from the study if there was evidence of other pregnancy complications, such as chronic hypertension, type 1 or type 2 diabetes mellitus, seizure disorder and renal or liver disease. All study participants neither consumed alcohol nor smoked and were from a low socioeconomic group.

The normotensive group consisted of pregnant women with no medical or obstetric complications. Pregnant women with eclampsia were excluded from the study. PE was diagnosed by an obstetrician who is also one of the investigators in this study (SSM) and PE has been discussed by us in a number of our earlier studies [45-47]. PE was defined by systolic and diastolic blood pressures greater than 140 and 90 mm Hg, respectively, with presence of proteinuria (>1+ or 300 mg/24 hrs) on a dipstick test and was confirmed by repeated recording of the blood pressure with an interval of 6 hrs. Blood pressure was recorded frequently, starting at enrollment and at every follow up visit, which occurred approximately once a month until delivery. Response: Yes the preceding statement may be deleted. The data provided are the blood pressure values at the time of delivery, that is, just before going to the labor room, to ensure that a similar time point was used for both groups to rule out the effect on blood pressure of stress due to labor. Treatment of preeclamptic women included antihypertensive drugs and arginine supplementation. In severe PE cases, magnesium sulphate was given intravenously. Gestational age was based on the day of last menstrual period and was confirmed by ultrasound. All women were routinely given iron and folic acid supplements as per the National Anemia Prophylaxis Program.

### Tissue collection and processing

Fresh placental tissues were obtained from normal and preeclamptic pregnancies immediately after delivery. Fetal membranes were trimmed off and small pieces were randomly cut out from the placental cotyledons. Tissue were rinsed in phosphate-buffered saline to wash off maternal and fetal blood, snap frozen in liquid nitrogen and stored at −80°C until assayed.

### Gene promoter methylation assay

**Figure 5:**
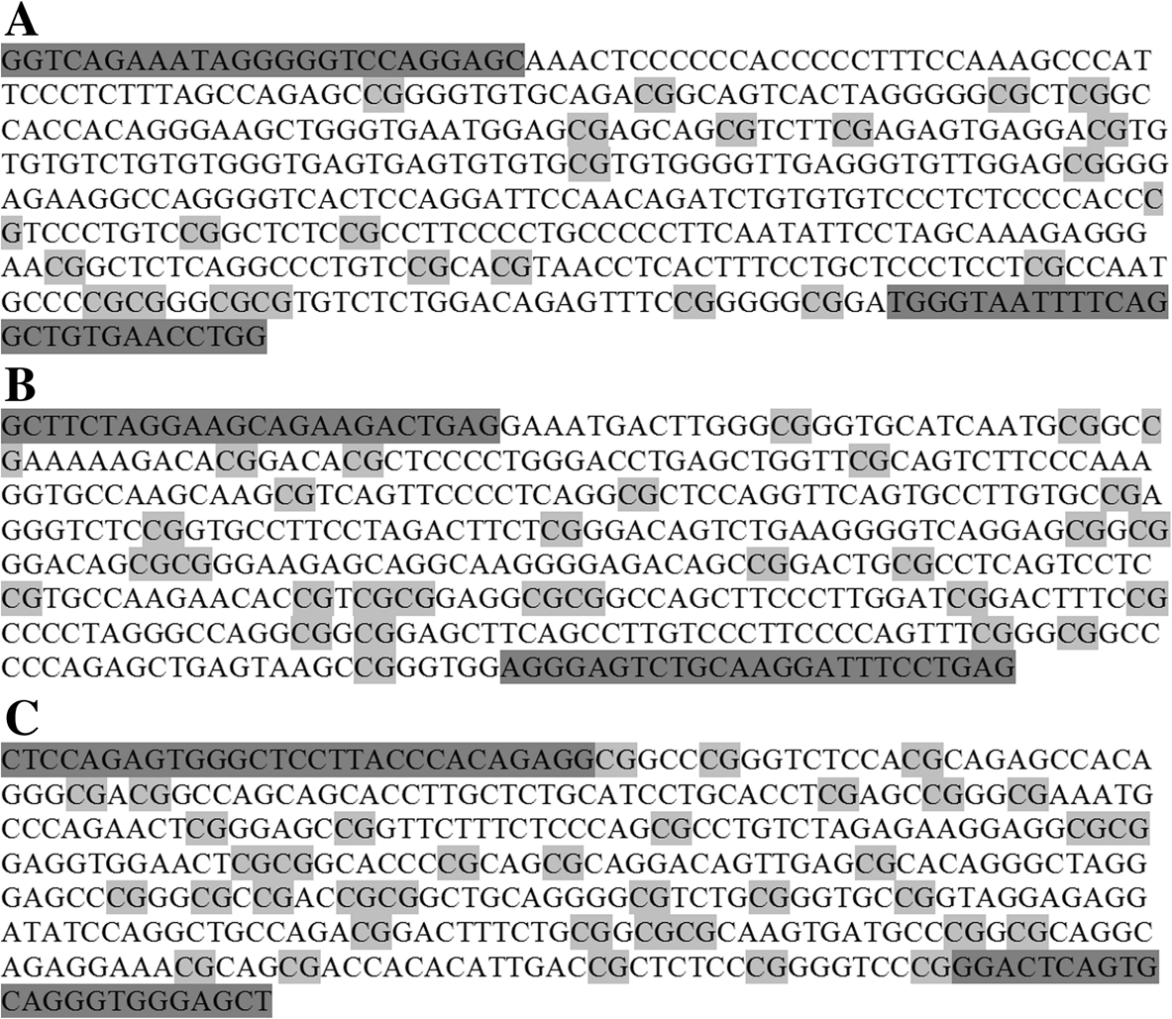
**Promoter sequences selected for analysis of CpG methylation of (A) *VEGF *(B) *FLT*-1 and (C) *KDR *genes.** Sequences highlighted in dark gray indicate primers. Sequences highlighted in light gray indicate CpG sites analyzed.

Genomic DNA was isolated from placental samples using the DNeasy Blood and Tissue kit (Cat No. 69504, Qiagen, Hilden, Germany) using the protocol supplied by the manufacturer. One microgram of purified genomic DNA was bisulfite-treated using the EZ DNA Methylation™ Kit (Cat No. D5006, Zymo Research, California, USA) as per the manufacturer’s instructions. The bisulfite-modified DNA was used for Sequenom MassARRAY EpiTYPING for gene-specific methylation analysis of *VEGF*, *FLT*-1, and *KDR*. In this technique, base-specific cleavage followed by MALDI-TOF mass spectrometry is used. The size ratio of the cleaved products provides quantitative methylation estimates for CpG sites within a target region. Genomic sequences for assay design were extracted from the UCSC genome browser (http://www.genome.ucsc.edu/). Primer pairs for amplification were designed using EpiDesigner web tool (http://www.epidesigner.com/). The promoter CpG island sequences used in this study for CpG methylation analysis of *VEGF* (Chromosome 6, 43737274 to 43737739), *FLT*-1 (Chromosome 13, 29067752 to 29068196) and *KDR* (Chromosome 4, 55991374 to 55991777) were selected using the UCSC genome browser and are given in Figure 5. For PCR amplification, a T7-promoter tag was added to the reverse primer for performing the *in vitro* transcription, and a 10-mer tag sequence was added to the forward primer to balance the PCR primer length. The primers are listed in Table 3.

**Table 3:** Primers used for promoter CpG analysis

**Primer code**	Primer sequence (5′-3′)*	**Amplicon length (base pairs)**	**Number of CpG sites analyzed**
VEGF_F1	aggaagagagTTAGAAATAGGGGGTTTAGGAGTAAAT	465	23
VEGF_R1	cagtaatacgactcactatagggagaaggctCCAAATTCACAACCTAAAAATTACCCA		
FLT1_F1	aggaagagagGTTTTTAGGAAGTAGAAGATTGAGG	444	30
FLT1_R1	cagtaatacgactcactatagggagaaggctCTCAAAAAATCCTTACAAACTCCCT		
KDR_F1	aggaagagagTTTTAGAGTGGGTTTTTTATTTATAGAGG	403	37
KDR_R1	cagtaatacgactcactatagggagaaggctAACTCCCACCCTACACTAAATCC		

* T7-promoter sequence tag -cagtaatacgactcactatagggagaaggct added to reverse primer.

10-mer sequence tag- aggaagagag added to the forward primer.

Bisulfite-treated genomic DNA was amplified using the designed primers. The thermal cycling conditions were as follows. For the *VEGF*- 1 cycle: 95°C for 15 minutes; 5 cycles: 95°C for 1 minutes, 62°C for 2 minutes, 72°C for 2 minutes; 32 cycles: 95°C for 1 minute, 62°C for 1 minute, 72°C for 1 minute then 72°C for 7 minutes. For the *FLT*-1 and *KDR*-1 cycle: 95°C for 15 minutes; 5 cycles: 95°C for 1 minute, 60°C for 2 minutes, 72°C for 2 minutes; 35 cycles: 95°C for 1 minutes, 60°C for 1 minute, 72°C for 1 minute then 72°C for 7 minute.

Following PCR amplification, *in vitro* transcription and T-cleavage assay was performed using MassCLEAVE™ Reagent Kit (Cat No. 10129, Sequenom). Unincorporated dinucleotide triphosphates were removed by shrimp alkaline phosphatase treatment. Typically, 2 μl of the PCR product was directly used as template for the *in vitro* transcription reaction. T7 RNA and DNA polymerase was used to incorporate thymidine triphosphate in the transcripts. In the same step, RNase-A was added to cleave the *in vitro* transcripts (T-cleavage assay). Samples were diluted with 20 μl of water. Conditioning of the phosphate backbone was done by adding 6 mg of Clean Resin before performing MALDI-TOF mass spectrometry (MS) analysis. For MS analysis RNase-A-treated product was robotically dispensed onto silicon matrix preloaded chips (SpectroCHIP Cat No. 10117, Sequenom), and the mass spectra were collected using a MassARRAY Compact MALDI-TOF (Sequenom). Methylation data were generated as β values between 0 and 1, indicating percentage methylation of the original template. The methylation ratios were generated from the spectra by the EpiTYPER software v1.0 (Sequenom, California, USA).

Samples that yielded data in greater than 70% for all CpG units within a promoter were passed for that sample/promoter pair. For each sample the methylation analysis was done in duplicates and sites showing more than 10% difference in methylation were excluded. Sites that were tagged as low mass or high mass by Epityper software were also excluded from the analysis. We attempted to predict the transcription factor binding sites in the *VEGF*, *FLT*-1 and *KDR* promoter regions using the transcription factor binding site predictor tool, PROMO (version 3.0.2) (http://alggen.lsi.upc.es/cgi-bin/promo_v3/promo/promoinit.cgi?dirDB=TF_8.3) [48,49].

### Extraction of total RNA, cDNA synthesis and quantitative real-time (RT)-PCR assays

Total RNA from placenta samples was isolated using the Trizol method and quantified by the Nanodrop (ND1000 v3.5.2) spectrophotometer. One microgram of total RNA was transcribed to cDNA using the High-Capacity cDNA reverse transcription Kit (Cat No. 4368814, Applied Biosystems, California, USA). RT-quantitative (q)-PCR for *VEGF*, *FLT*-1, *KDR* mRNAs, and 18S rRNA were performed with the TaqMan Universal PCR Master Mix (Cat No. 4324018, Applied Biosystems, California, USA) using the Applied Biosystems 7500 FAST system. The relative expression level of the gene of interest was examined with respect to 18S rRNA to normalize for variation in the quality of RNA and the amount of input cDNA. The RT-PCR reactions for each gene were performed in duplicate. To analyze the RT-PCR results, the average cycle number (Ct) of the reaction when it crossed a threshold valued was determined for each reaction. Differences in Ct (ΔCt) between 18S and the targeted gene were calculated by subtracting the Ct of the targeted gene from Ct of 18S. Relative expression levels of genes were calculated and expressed as 2^ΔCt^. The following TaqMan® Gene Expression Assays (Applied Biosystems, California, USA) were used in this study: 18S RNA (Hs99999901_s1); *VEGF* (Hs00900058_m1); *FLT*-1 (Hs01052936_m1); *KDR* (Hs00176676_m1).

### Statistical analysis

Data were analyzed using SPSS/PC + package (Version 20.0, Chicago, IL, USA). Values are reported as mean ± SD (demographic characters) or mean ± standard error (SE) (gene expression and methylation studies). Mean values of the estimates were compared using one-way ANOVA and the post hoc least significant difference (LSD) test at conventional levels of significance (*P* <0.05). Skewed variables were transformed to normality using log to the base 10. The extent of the linear relationship between several variables was studied using bivariate correlation analysis.

## Abbreviations

ANOVA: Analysis of variance; Ct: Average cycle number; FLT-1: fms-like tyrosine kinase-1; KDR: Kinase insert domain containing receptor; LSD: Least significant difference; MS: Mass spectrometry; PCR: Polymerase chain reaction; PE: Preeclampsia; RT-PCR: Real-time polymerase chain reaction; SE: Standard error; VEGF: Vascular endothelial growth factor; VEGFR: Vascular endothelial growth factor receptor.

## Competing interests

The authors declare that they have no competing interests.

## Authors’ contributions

SRJ and SSM conceived and designed the experiments. DPS, USR, AAJ, PMCG and GRC performed the experiments. DPS, USR, AAJ, PMCG, SRJ and GRC analyzed the data. SSM, AAH and GRC contributed reagents/materials/analysis tools. PMCG, DPS, AAJ and SRJ wrote the paper. All authors read and approved the final manuscript.

## Supplementary Material

Additional file 1 **(A) Mean percent methylation at each CpG site in the VEGF promoter.** *P<0.05, ** P <0.01 as compared to control. PE, preeclampsia; (B) Mean percent methylation at each CpG site in the FLT-1 promoter. * P <0.05, ** P <0.01 as compared to control; @ P <0.05 as compared to Term PE. PE, preeclampsia; (C) Mean percent methylation at each CpG site in the KDR promoter. * P <0.05 as compared to control. PE, preeclampsia. Click here for file

Additional file 2 Transcription factor binding sites predicted in the promoter region of (a) VEGF (b) FLT-1 and (c) KDR genes. Click here for file
