# Supplementary material for: Differential placental methylation and expression of VEGF, FLT-1 and KDR genes in human term and preterm preeclampsia
Source: Clin Epigenetics. 2013 Apr 26;5(1):6. doi: 10.1186/1868-7083-5-6 (PMC3640948; doi:10.1186/1868-7083-5-6)
Supplement: Additional file 1 — (A) Mean percent methylation at each CpG site in the VEGF promoter. *P<0.05, ** P <0.01 as compared to control. PE, preeclampsia; (B) Mean percent methylation at each CpG site in the FLT-1 promoter. * P <0.05, ** P <0.01 as compared to control; @ P <0.05 as compared to Term PE. PE, preeclampsia; (C) Mean percent methylation at each CpG site in the KDR promoter. * P <0.05 as compared to control. PE, preeclampsia. [file 1868-7083-5-6-S1.doc]

1. **Mean percent methylation at each CpG site in the *VEGF* promoter**

|  | **Percent methylation (%)** | | |
| --- | --- | --- | --- |
| **CpG site** | Mean ± S.E. | | |
|  | **Normotensive** | **Term PE** | **Preterm PE** |
| **CpG - 1** | 3.0 ± 0.2 | 2.8 ± 0.1 | 3.0 ± 0.3 |
| **CpG - 3.4** | 6.4 ± 0.4 | 6.1 ± 0.3 | 5.7 ± 0.3 |
| **CpG - 6.7** | 4.2 ± 0.2 | 3.7 ± 0.2 | 3.5 ± 0.4* |
| **CpG - 8** | 7.1 ± 0.1 | 6.8 ± 0.1 | 6.4 ± 0.2** |
| **CpG - 9** | 5.9 ± 0.1 | 5.6 ± 0.1 | 5.7 ± 0.3 |
| **CpG - 14** | 4.2 ± 0.3 | 4.9 ± 0.3 | 5.2 ± 0.4* |
| **CpG - 15** | 3.2 ± 0.1 | 3.2 ± 0.1 | 3.0 ± 0.2 |
| **CpG - 22.23** | 6.1 ± 0.2 | 6.1 ± 0.3 | 5.8 ± 0.2 |
| **Mean Promoter** | 5.1 ± 0.1 | 4.9 ± 0.1 | 4.7± 0.2* |

*p<0.05, **p<0.01 as compared to control

1. **Mean percent methylation at each CpG site in the *FLT-1* promoter**

|  | **Percent methylation (%)** | | |
| --- | --- | --- | --- |
| **CpG site** | Mean ± S.E. | | |
|  | **Normotensive** | **Term PE** | **Preterm PE** |
| **CpG - 1** | 2.9 ± 0.1 | 3.2 ± 0.2 | 3.2 ± 0.1 |
| **CpG - 5** | 1.0 ± 0.1 | 1.0 ± 0.1 | 0.9 ± 0.1 |
| **CpG - 6** | 1.3 ± 0.1 | 1.1 ± 0.1 | 1.2 ± 0.2 |
| **CpG - 7** | 4.6 ± 0.5 | 3.8 ± 0.5 | 5.0 ± 0.8 |
| **CpG - 8** | 0.9 ± 0.2 | 1.1 ± 0.2 | 1.0 ± 0.2 |
| **CpG - 9** | 4.7 ± 0.2 | 4.6 ± 0.1 | 4.5 ± 0.1 |
| **CpG - 10** | 5.7 ± 0.2 | 5.3 ± 0.1 | 5.5 ± 0.1 |
| **CpG - 11** | 2.9 ± 0.1 | 2.7 ± 0.2 | 2.8 ± 0.2 |
| **CpG – 12.13** | 1.0 ± 0.1 | 0.9 ± 0.1 | 1.1 ± 0.1 |
| **CpG – 14.15** | 0.2 ± 0.04 | 0.1 ± 0.04 | 0.2 ± 0.1 |
| **CpG - 16** | 1.6 ± 0.2 | 0.8 ± 0.2** | 1.2 ± 0.2 |
| **CpG - 17** | 3.2 ± 0.1 | 3.2 ± 0.1 | 2.7 ± 0.1***@** |
| **CpG - 18** | 1.4 ± 0.1 | 1.3 ± 0.1 | 1.6 ± 0.1 |
| **CpG – 19.20.21** | 2.9 ± 0.1 | 2.8 ± 0.1 | 2.9 ± 0.2 |
| **CpG – 22.23** | 3.4 ± 0.2 | 3.4 ± 0.2 | 3.5 ± 0.3 |
| **CpG - 24** | 1.1 ± 0.1 | 0.7 ± 0.1* | 0.6 ± 0.1* |
| **CpG - 25** | 2.1 ± 0.05 | 2.2 ± 0.05 | 1.9 ± 0.1 |
| **CpG – 26.27** | 1.0 ± 0.1 | 0.9 ± 0.1 | 1.1 ± 0.1 |
| **CpG – 28.29** | 4.2 ± 0.2 | 4.8 ± 0.3 | 4.5 ± 0.3 |
| **CpG – 30** | 0.3 ± 0.06 | 0.3 ± 0.05 | 0.6 ± 0.1 |
| **Mean Promoter** | 2.3 ± 0.05 | 2.2 ± 0.05 | 2.3 ± 0.07 |

*p<0.05, **p<0.01 as compared to control; **@**p<0.05 as compared to Term PE

1. **Mean percent methylation at each CpG site in the *KDR* promoter**

|  | **Percent methylation (%)** | | |
| --- | --- | --- | --- |
| **CpG site** | Mean ± S.E. | | |
|  | **Normotensive** | **Term PE** | **Preterm PE** |
| **CpG – 1.2** | 15.1 ± 1.2 | 14.2 ± 1.2 | 13.0 ± 1.2 |
| **CpG - 4** | 10.9 ± 1.4 | 14.3 ± 1.5 | 12.8 ± 1.5 |
| **CpG - 5** | 6.3 ± 0.9 | 8.4 ± 1.1 | 8.4 ± 1.2 |
| **CpG - 6** | 23.4 ± 2.2 | 23.5 ± 2.2 | 23.3 ± 2.2 |
| **CpG – 7.8** | 10.3 ± 1.6 | 13.2 ± 1.8 | 14.3 ± 1.7 |
| **CpG - 9** | 2.1 ± 0.6 | 3.0 ± 1.0 | 2.2 ± 0.4 |
| **CpG - 10** | 5.4 ± 0.4 | 7.0 ± 0.9 | 5.9 ± 0.6 |
| **CpG - 11** | 5.8 ± 0.6 | 7.3 ± 0.8 | 6.6 ± 0.8 |
| **CpG – 12.13** | 4.9 ± 0.7 | 7.6 ± 1.1* | 8.2 ± 1.1* |
| **CpG – 14.15** | 4.0 ± 0.6 | 4.8 ± 0.7 | 4.5 ± 0.7 |
| **CpG - 16** | 8.6 ± 0.8 | 10.8 ± 1.3 | 8.6 ± 0.9 |
| **CpG - 17** | 2.0 ± 0.5 | 2.5 ± 0.7 | 3.2 ± 0.8 |
| **CpG - 18** | 2.0 ± 0.5 | 2.5 ± 0.7 | 3.2 ± 0.8 |
| **CpG – 19.20.21** | 10.2 ± 1.2 | 11.7 ± 1.4 | 11.2.0 ± 1.4 |
| **CpG – 22.23** | 12.3 ± 1.1 | 14.5 ± 1.7 | 13.6 ± 1.4 |
| **CpG – 24.25.26** | 14.5 ± 1.1 | 16.2 ± 1.4 | 15.4 ± 1.4 |
| **CpG – 28.29.30** | 13.8 ± 1.4 | 16.3 ± 1.5 | 14.4 ± 1.4 |
| **CpG – 31.32** | 12.3 ± 1.1 | 14.5 ± 1.7 | 13.6 ± 1.4 |
| **CpG – 35.36.37** | 20.2 ± 0.8 | 20.1 ± 0.9 | 20.1 ± 0.9 |
| **Mean Promoter** | 9.9 ± 0.8 | 11.5 ± 1.0 | 10.7 ± 0.9 |

*p<0.05 as compared to control
