# Supplementary material for: Differential placental methylation and expression of VEGF, FLT-1 and KDR genes in human term and preterm preeclampsia
Source: Clin Epigenetics. 2013 Apr 26;5(1):6. doi: 10.1186/1868-7083-5-6 (PMC3640948; doi:10.1186/1868-7083-5-6)
Supplement: Additional file 2 — Transcription factor binding sites predicted in the promoter region of (a) VEGF (b) FLT-1 and (c) KDR genes. [file 1868-7083-5-6-S2.doc]

**Transcription factor binding sites predicted in the promoter region of (a) *VEGF* (b) *FLT*-1 and (c) *KDR* genes.**

**(a)**

GGTCAGAAATAGGGGGTCCAGGAGCAAACTCCCCCCACCCCCTTTCCAAAGCCCATTCCCTCTTTAGCCAGAGCCG

AP-1

**1**

**__________**

GGGTGTGCAGA**CGGCAGTCA**CTAGGGGGCGCTCGGCCACCACAGGGAAGCTGGGTGAATGGAGCGAGCAGCGT **_________**

NF-1

**2**

**3**

**4**

**5**

**6**

CTTCGAGAGTGAGGACGTGTGTGTCTGTGTGGGTGAGTGAGTGTGTGCGTGTGGGGTTGAGGGTG**TTGGAGCG**G

**7**

**8**

**9**

**10**

GGAGAAGGCCAGGGGTCACTCCAGGATTCCAACAGATCTGTGTGTCCCTCTCCCCACCCGTCCCTGTCCGGCTCTC

**11**

**12**

AP-2 alpha A

HIF-1

**______ _________ ___**

CGCCTTCCCCTGCCCCCTTCAATATTCCTAGCAAAGAGGGAACGGCTC**TCAGGC**CCTG**TCCGCACGT**AACCTCA**CTT**

STAT1 beta

**13**

**16**

**15**

Sp1

STAT4

**14**

**_______** **______** **___________**

**TCCTGCT**CCCTCCTCGCCAATGCCCCGCGGGCGCGTGTCTCTGGACAGA**GTTTCC**G**GGGGCGGATG**GGTAATTTTC

**17**

**18 19 20 21**

**22 23**

AGGCTGTGAACCTGG

**(b)**

AP-1

**2**

STAT4

STAT1beta

_________ GCTTCTAGGAAGCAGAAGA**CTGAGGAAATGACTTGGG**CGGGTGCATCAATGCGGCCGAAAAAGACACGGACACG

**1**

**3**

**4**

**5**

c-Jun

________

CTCCCCTGGGACCTGAGCTGGTTCGCAGTCTTCCCAAAGGTGCCAAGCA**AGCGTCA**GTTCCCCTCAGGCGCTCCAG

**6**

**7**

**8**

GTTCAGTGCCTTGTGCCGAGGGTCTCCGGTGCCTTCCTAGACTTCTCGGGACAGTCTGAAGGGGTCAGGAGCGGC

**9**

**10**

**11**

**12 13**

AP-2alpha A

______

GGGACAGCGCGGGAAGA**GCAGG**CAAGGGGAGACAGCCGGACTGCGCCTCAGTCCTCCGTGCCAAGAACACCGT

**14 15**

**16**

**17**

**18**

**19**

CGCGGAGGCGCGGCCAGCTTCCCTTGGATCGGACTTTCCGCCCCTAGGGCCAGGCGGCGGAGCTTCAGCCTTGTC

STAT1beta

**20 21**

**22 23**

**24**

**25**

**26 27**

__________

CCTTCCCCAGTTTCGGGCGGCCCCCAGAGCTGAGTAAGCCGGGTGGAGGGAGTCTGCAAGG**ATTTCCTGAG**

**28 29**

**30**

STAT4

**(c)**

CTCCAGAGTGGGCTCCTTACCCACAGAGGCGGCCCGGGTCTCCACGCAGAGCCACAGGGCGACGGCCAGCAGCA

**1**

**2**

**3**

**4**

**5**

CCTTGCTCTGCATCCTGCACCTCGAGCCGGGCGAAATGCCCAGAACTCGGGAGCCGGTTCTTTCTCCCAGCGCCTG

STAT4

**6**

**8**

**9**

**10**

**11**

______

TCTAGAGAAGGAGGCGCGGAGGT**GGAACT**CGCGGCACCCCGCAGCGCAGGACAGTTGAGCGCACAGGGCTAGG

GATA-1

**12 13**

**14 15**

**16**

**17**

**18**

______ ______

GAGCCCGGGCGCCGACCGCGGCTGCAGGGGCGTCTGCGGGTGCCGGTAGGAG**AGGATATCCAGG**CTGCCAGAC

**23**

**25**

AP-2 alpha A

**19**

**20 21**

**21 22**

STAT4

_______ ______ GGACTTTCTGCGGCGCGCAAGTGATGCCCGGC**GCAGGC**AGA**GGAAAC**GCAGCGACCACACATTGACCGCTCTCC

**26**

**27 28 29**

**30 31**

**32 33**

**34**

CGGGGTCCCGGGACTCAGTGCAGGGTGGGAGCT

**35**

Sequences highlighted in dark gray indicate primers. Sequences highlighted in light gray indicate CpG sites analysed. Transcription factor binding sites are indicated in bold font.
